# Supplementary material for: Impact of Temperature and Oxygen Availability on Gene Expression Patterns of Mycobacterium ulcerans
Source: Microbiol Spectr. 2023 Mar 13;11(2):e04968-22. doi: 10.1128/spectrum.04968-22 (PMC10100886; doi:10.1128/spectrum.04968-22)
Supplement: Supplemental file 3 — Supplemental material. Download spectrum.04968-22-s0003.pdf, PDF file, 1.1 MB [file spectrum.04968-22-s0003.pdf]

Supplementary Material

Supplemental Figures

Supplemental Figure 1. Heat map of entire statistically differentially regulated gene transcripts across treatments and timepoints.

Supplemental Table 1. Fold Change in Gene expression according to Treatment. Listed in the table include the treatments, gene name or RefSeq original locus tag, genome region, FDR p-value, gene annotation, former locus tag also listed in RefSeq associated with the reference genomes, and functional hierarchies 1-3 identified through databases listed in Materials and Methods.

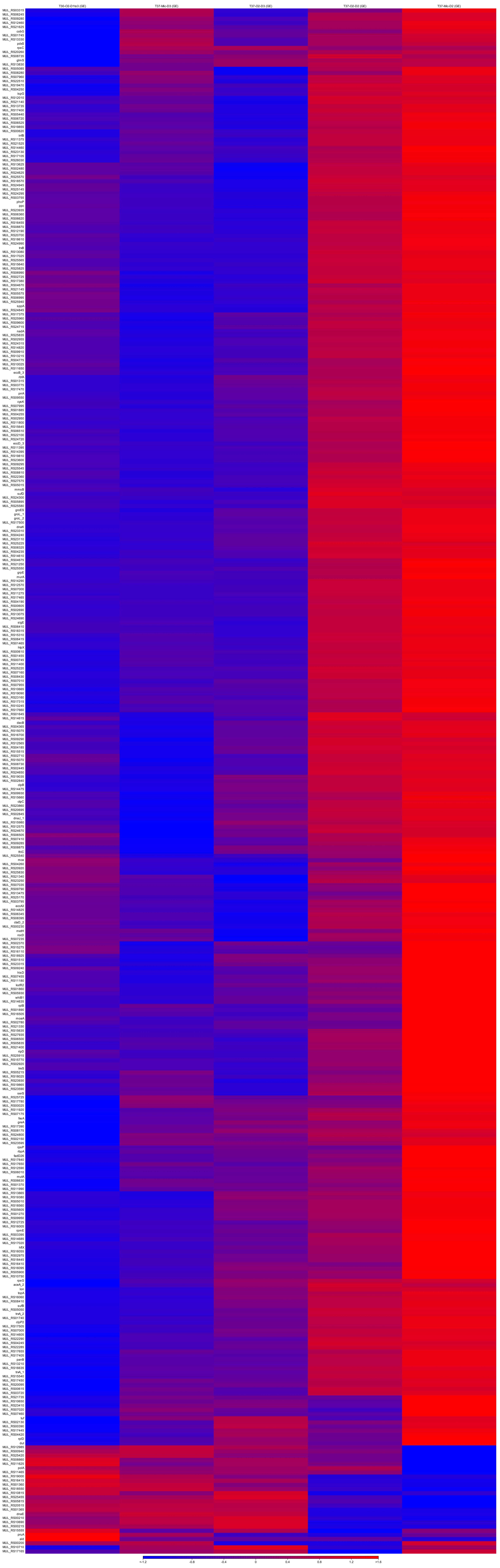

T30-O2-D1to3 (GE)
T37-Mo-D3 (GE)
T37-O2-D3 (GE)
T37-O2-D2 (GE)
T37-Mo-D2 (GE)
<-1.2
-0.8
-0.4
0
0.4
0.8
1.2
>1.6
